# Native ESI-MS and Collision-Induced Unfolding (CIU) of the Complex between Bacterial Elongation Factor-Tu and the Antibiotic Enacyloxin IIa

**DOI:** 10.1021/jasms.4c00087

**Published:** 2024-06-03

**Authors:** Cameron Baines, Jacob Sargeant, Christopher D. Fage, Hannah Pugh, Lona M. Alkhalaf, Gregory L. Challis, Neil J. Oldham

**Affiliations:** † School of Chemistry, University of Nottingham, University Park, Nottingham NG7 2RD, United Kingdom; ‡ Department of Chemistry, University of Warwick, Coventry CV4 7AL, United Kingdom; § Warwick Integrative Synthetic Biology Centre, University of Warwick, Coventry CV4 7AL, United Kingdom; ∥ Department of Biochemistry and Molecular Biology, Biomedicine Discovery Institute, Monash University, Clayton, Victoria 3800, Australia; ⊥ ARC Centre of Excellence for Innovations in Peptide and Protein Science, Monash University, Clayton, Victoria 3800, Australia

## Abstract

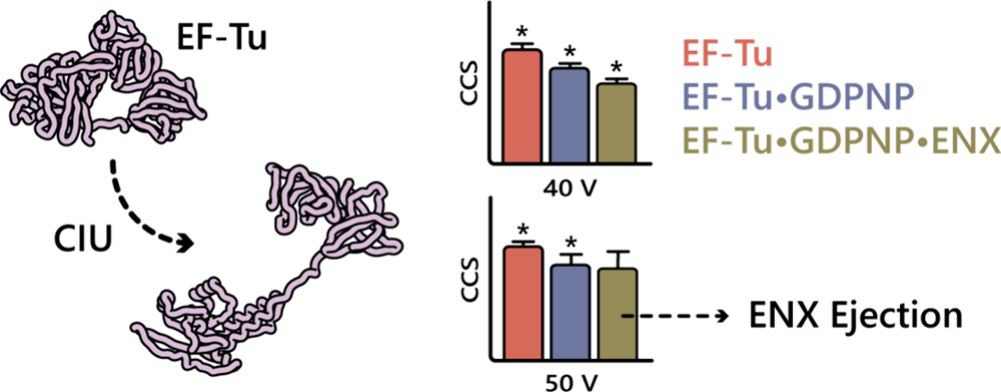

Collision-induced unfolding (CIU) of protein ions, monitored by ion mobility-mass spectrometry, can be used to assess the stability of their compact gas-phase fold and hence provide structural information. The bacterial elongation factor EF-Tu, a key protein for mRNA translation in prokaryotes and hence a promising antibiotic target, has been studied by CIU. The major [M + 12H]^12+^ ion of EF-Tu unfolded in collision with Ar atoms between 40 and 50 V, corresponding to an *E*_lab_ energy of 480–500 eV. Binding of the cofactor analogue GDPNP and the antibiotic enacyloxin IIa stabilized the compact fold of EF-Tu, although dissociation of the latter from the complex diminished its stabilizing effect at higher collision energies. Molecular dynamics simulations of the [M + 12H]^12+^ EF-Tu ion showed similar qualitative behavior to the experimental results.

## Introduction

Native electrospray ionization-mass spectrometry (ESI-MS) and related techniques are finding wide application in structural biology.^1^ These methods exploit the soft ionization properties of ESI and further rely upon (i) the use of aqueous ESI solvents, often containing volatile salts such as ammonium acetate, and (ii) careful control of voltages and pressures in the mass spectrometer to transmit large biomacromolecular ions through the optics of the instrument with minimal structural perturbation.^2^ Under these conditions, it is possible to maintain noncovalent interactions, such as those seen in multiprotein complexes or between proteins and small molecule ligands, such as enzyme cofactors, substrates, and inhibitors. From such measurements, important information on stoichiometry and apparent binding affinity may be readily derived. The addition of ion mobility spectrometry (IMS) further provides information on the size of such complexes through determination of the collisional cross section (CCS) of the ions.

Collision-induced unfolding (CIU) is a method for studying the unfolding of (usually) protein ions in the gas-phase using programmed collisional activation prior to ion mobility analysis.^3^ Accelerating ions into a neutral collision gas, such as argon, transfers kinetic energy into internal energy within the compact protein ions and causes them to unfold. The resulting increase in CCS, as measured by IMS, may be determined as a function of applied energy and utilized as a measure of the stability of the compact fold. Initial experiments into collisional activation and analyses via IMS involved the direct injection of ions into the pressurized drift cell, in which collision with the neutral buffer gas results in *in situ* fragmentation.^4^ This is in contrast to the system used in this study, where ions undergo collisional activation in a trap cell prior to entry into the drift cell.

The groups of Jarrold and Clemmer were pioneers in monitoring protein ion unfolding,^5,6^ although the term CIU was not coined until later, where it was used by us to demonstrate the stabilization of protein structure upon ligand binding.^7^ Brandon Ruotolo, in whose honor this special issue of JASMS is dedicated, has led the field in the application of CIU to studying protein stability. His group has applied CIU to systems including protein–ligand structural stabilization,^8,9^ the effects of anion adduction on structural stability,^10^ determination of folded domains in a protein structure,^11^ the characterization of disulfide bonding patterns and glycoforms in antibodies,^10,12^ assessment of bispecific antibodies,^13^ as well as heat stressed antibodies,^14^ and distinguishing competitive vs allosteric kinase inhibitors.^15^

Our most recent work has utilized CIU to probe residues important in maintaining compact gas-phase protein structure in an acyl carrier protein and the model protein ubiquitin using alanine scanning and chemical modification, respectively.^16,17^ By selective modification of residues and measurement of the resulting effects on unfolding, it is possible to deduce whether those residues are involved in stabilizing intramolecular interactions. In the work reported here, we use CIU to study the effects of cofactor and antibiotic binding on the bacterial elongation factor EF-Tu, a promising antimicrobial target.

EF-Tu is a ubiquitous, 394-residue G-protein made up of three distinct domains: one GTP/GDP binding domain (1–200) and two oligonucleotide binding domains (208–295 and 298–394).^18^ The role of EF-Tu is to deliver aminoacyl-tRNA to the A site of the ribosome, thus facilitating mRNA translation.^19^ It does this by utilizing induced GTP hydrolysis to drive a conformational change which enables release of the protein from the ribosome, while cognate aminoacyl-tRNA remains.^20^ This GTP hydrolysis occurs at a rate strongly dependent on cognate codon:anticodon recognition.^21^ As EF-Tu plays such a vital role in translation, it is one of the most highly conserved proteins in prokaryotes and is an attractive antimicrobial target.^22^

EF-Tu was classically thought to be a two-conformation protein, with a GDP-bound “open” state alongside the GTP-bound “closed” state. However, it is now known that EF-Tu exists in solution in a range of conformations within these two extremes. Experimental FRET (fluorescence resonance energy transfer) microscopy and crystallography have demonstrated GTP-bound EF-Tu can adopt a conformation much closer to the open state, and conformations approaching the theoretical closed state are only reached upon ribosome binding.^23^

Several antibiotics have been discovered that target EF-Tu, collectively referred to as elfamycins. These are generally divided into two groups, based on their mechanism of action. The first type prevents EF-Tu dissociating from the ribosome and includes kirromycin (KIR) and enacyloxin IIa (ENX), while the second type inhibits aminoacyl-tRNA from binding to the enzyme and includes pulvomycin (PUL) and GE2270A (GEA).^24^

In this study we use CIU and molecular dynamics (MD) simulations to examine the role of nucleotide cofactor and ENX binding on the stability of compact EF-Tu in the gas phase. We show that the binding of GDPNP (a nonhydrolyzable analogue of GTP) and ENX increases the energy required to induce unfolding and that the effect of ENX is modulated at higher energies by concurrent dissociation from the protein.

## Experimental Section

### Sample Preparation

*Escherichia coli* EF-Tu was expressed in *E. coli* (see Supporting Information), purified using Ni-NTA affinity chromatography, theHis_6_-tag cleaved,^25^ and stored at −80 °C. Aliquots (1 μL) were thawed on ice prior to dilution with either 4 μL of 50 mM aqueous ammonium acetate (Fisher Scientific) or 2 μL of 5 mM 5′-guanylyl imidodiphosphate (GDPNP, Sigma-Aldrich) and 2 μL of 5 mM magnesium acetate tetrahydrate (Sigma-Aldrich) in 50 mM ammonium acetate. Samples were then incubated on ice for 10 min, during which Zeba Micro Spin Desalting Columns (7K MWCO, 75 μL working volume, Thermo Fisher Scientific) were prepared by addition of 50 μL of 50 mM ammonium acetate followed by centrifugation at 4 °C, 1000 RCF for 1 min, repeated four times. After incubation, the EF-Tu samples were added to the preprepared desalting column and subjected to centrifugation at 4 °C, 1000 RCF for 2 min, and the eluent was stored on ice.

### Native ESI-MS

All experiments were performed on a G1 SYPNAPT HDMS (Waters Corporation) operating in positive ion mode, using glass emitter tips fitted with platinum wire (Sigma-Aldrich). Emitter tips were pulled in-house from borosilicate glass capillary tubes (0.8/1.0 mm internal/external diameter respectively, Hirschmann) using a Flaming/Brown P-97 micropipette puller (Sutter Instruments). This resulted in tips with an approximately 1 cm taper leading to a 0.3–0.6 μm internal diameter orifice. For use in the mass spectrometer, desalted EF-Tu aliquots (see above) were further diluted twofold in 50 mM ammonium acetate or 50 mM ammonium acetate containing 20 μM ENX, resulting in working protein concentrations of approximately 7.5 μM with ENX at 5 μM, where added. Proteins for use in ligand-bound experiments were purposefully incubated with substoichiometric concentrations of ligand to give samples containing apo protein, GDPNP-bound protein, and GDPNP·ENX-bound protein.

Samples were loaded into nESI emitter tips using GELoader pipet tips (Eppendorf), and the emitter assembly was loaded into the mass spectrometer. For each experiment, the *x, y, z* position of the emitter assembly and the applied capillary voltage were optimized to give the best signal (capillary voltage, 1.3–2.2 kV). All other instrument parameters were kept constant when operating in standard Q-TOF mode. Resulting total ion chromatograms and mass spectra were processed using MassLynx 4.1 software (Waters Corporation). Instrument operating parameters can be found in supplementary Table 1.

### Ion Mobility-Mass Spectrometry

The Synapt TWIMS cell was calibrated for collisional cross section (CCS) measurement using denatured myoglobin, native cytochrome C, and native ubiquitin arrival times. The 12^+^ EF-Tu complex charge state was isolated using the in-built quadrupole analyzer and subjected to incremental increases in collision energy (20–60 V in 1 V steps, followed by 42–44 V in 0.5 V steps). Arrival time distributions were extracted for each of the three protein species peaks in MassLynx 4.1 at each collision energy. CIUSuite2 was used to visualize arrive time distributions, generate CIU fingerprint plots, calculate CIU_50_, and compile data into the CSV format for further analyses.^26^ Conversion of TWIMS arrival time distributions to calibrated ^TW^CCS_N2→He_ was performed as outlined by Ruotolo et al.,^27^ with the empirically derived constant being 1.41 (Figure S1) and nomenclature as proposed by Gabelica et al.^28^ Sigmoid curves fit to calculated ^TW^CCS_N2→He_ data allowed the continual extraction of , where  represents the percentage of the protein population that has undergone the identified unfolding event. All IMS-MS experiments were performed with quintuple repeats. Instrument operating parameters can be found in supplementary Table 1.

### Molecular Dynamics Simulations

All molecular dynamics simulations were performed CPU-bound on an AMD Windows 11 based computer, equipped with a 3.8 GHz 8-core CPU using GROMACS 2020.1 running on Ubuntu 20.04.3 LTS.^29^ All simulations were performed in the CHARMM36 force field.^30−32^ EF-Tu input structures 2BVN and 1EFC were taken from the RCSB Protein DataBank and stripped of bound ligands, ions, and water for apo simulations. For all holo simulations, standalone ligand (GDP, GDPNP, enacyloxin IIa, Mg^2+^) topology files were produced using CGenFF before manual merging of individual ligands into their respective protein structural and topological files.^32^ In order to replicate the charge state of the protein studied experimentally, a python-based toolkit, ChargePlacer, was used to reproducibly assign protons to chargeable sites to generate the net 12^+^ charge state studied (Table S2, Figure S6).^16^ In brief, ChargePlacer randomly protonates chargeable sites (aspartic acid, glutamic acid, lysine, arginine, histidine, and the N- or C-terminal residues) to give the desired charge state, after which the energy of the protonated sequence is calculated. If this minimum energy is lower than the current minimum, the protonated sequence is used to reseed the charge placement algorithm and the process repeated until a stable energy minimum is reached. Upon benchmarking the system (Figure S6), the resulting most observed minimized proton sequence was used within the molecular dynamics simulations. Source code is available for ChargePlacer at https://github.com/jbellamycarter/chargePlacer.

Gas-phase simulations were performed on four structures; open apo-EF-Tu, open holo-EF-Tu, closed apo-EF-Tu, and closed holo-EF-Tu. All simulations were performed in triplicate. Protein/protein complexes were centered in a large cubic 900 nm^3^ bounding box and subject to energy minimization via steepest descent for up to 10 000 steps. The minimized structures were then equilibrated for 50 ps at 298 K using H-bonds as LINCS constraints (iterations = 1, order = 4). For full production runs, this 50 ps simulation was continued for a further 5 ns. These consisted of a 1 ns linear thermal gradient from 298 to 950 K followed by 4 ns maintained at 950 K. To cope with the high energy imparted on the system, the simulation step size was reduced to 1 fs to increase simulation stability.

All simulation trajectories were analyzed through built-in GROMACS packages, calculating RMSD, ligand–protein center of mass distances, and SASA (gmx rms, gmx pairdist, and gmx SASA respectively). Conversions of GROMACS XTC trajectories to PDB formats were performed with gmx trjconv skipping every other time step. Projection approximation CCSs were calculated from the PDB files using the CCS_CALC_ function within DriftScope 2.0 (Waters Corporation) which required a sub-2 GB file to process, hence skipping every other time step during the conversion process. CCS_PA_ was calculated with a gas collision radius of 1.4, and then resulting data multiplied by an empirical value of 1.14 to give a corrected value, CCS_CALC_, which can be compared to the experimentally derived ^TW^CCS_N2→He_ values.

## Results and Discussion

### Native ESI-MS of EF-Tu

**Figure 1:**
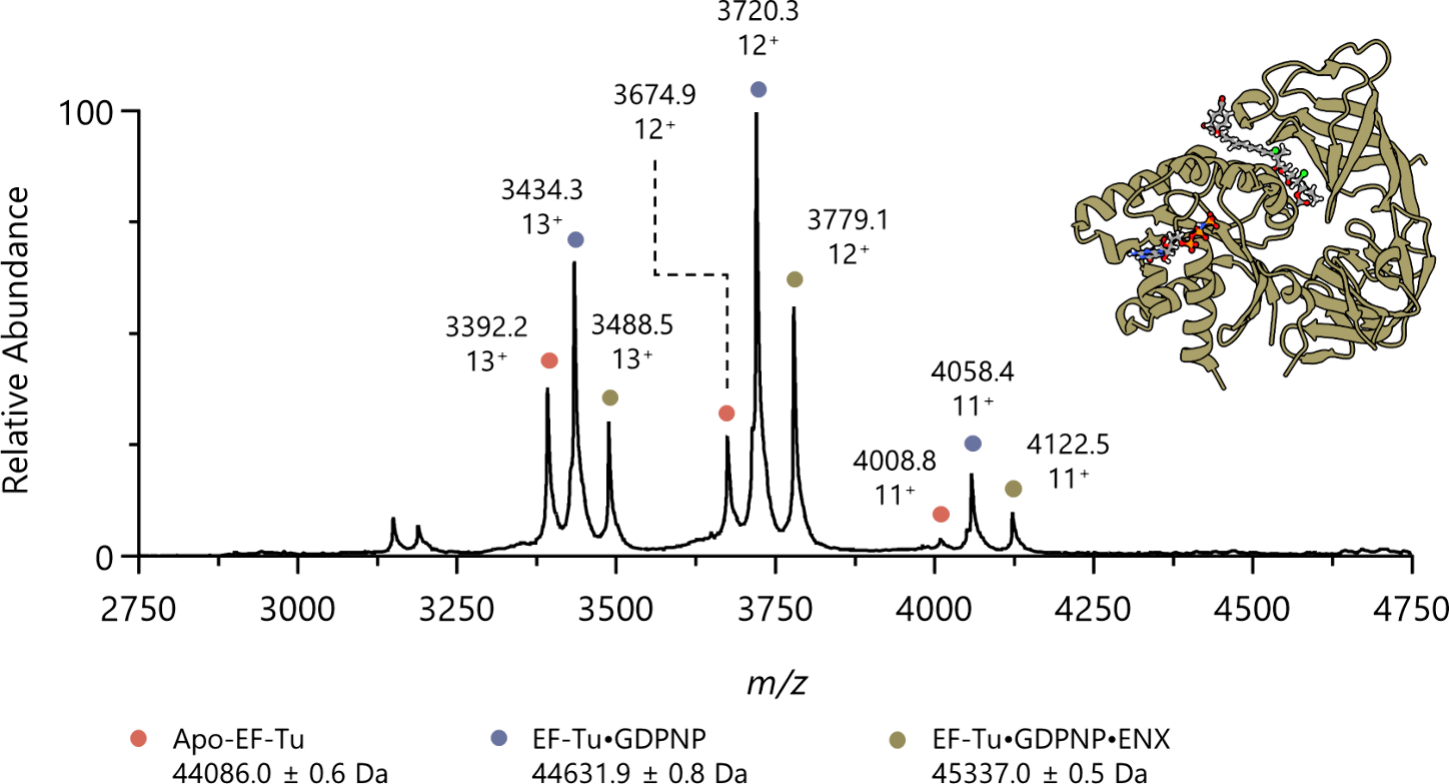
Native mass spectrum of *E. coli* EF-Tu. Nano-ESI of the protein complex resulted in three principal charge states: [M + 11H]^11+^, [M + 12H]^12+^, and [M + 12H]^13+^, with [M + 12H]^12+^ being the dominant charge state. Within each charge state, three protein species were observed: apo-EF-Tu (orange), EF-Tu·GDPNP (blue), and EF-Tu·GDPNP·ENX (green). Average deconvoluted masses of each species are shown along with an overlaid cartoon representation of the EF-Tu·GDPNP·ENX complex (PDB: 2BVN).

The ESI spectrum of EF-Tu, using the instrumental conditions described in the materials and methods section, exhibited three principal charge states: [M + 11H]^11+^, [M + 12H]^12+^, and [M + 12H]^13+^. EF-Tu was observed to bind GDPNP and GDPNP + ENX, and the deliberate addition of substoichiometric amounts of GDPNP and ENX yielded apo-EF-Tu, EF-Tu·GDPNP, and EF-Tu·GDPNP·ENX ions in the same spectrum (Figure 1). Upon quadrupole isolation and collision-induced dissociation (CID) of the [M + 12H]^12+^ (major) charge state of the EF-Tu·GDPNP·ENX complex, ENX was ejected in both neutral and ionized forms, as evidenced by the appearance of EF-Tu·GDPNP ions with [M + 12H]^12+^ and [M + 11H]^11+^ charge states (Figure S2). The collisionally activated ions were seen to have a reduced *m*/*z* compared to the native (Figure 1), likely due to the removal of protein-bound residual salts and solvent during activation.^33^ The presence of ENX^+^ ions in the low *m*/*z* region of the spectrum was further evidence of a proportion of ENX dissociating with a charge. No dissociation of GDPNP or GDP was observed under IM-MS conditions, but ejection of GDP and GDPNP was observed under CID activation at relatively high energies in MS-only mode, with the latter showing a lower propensity to dissociate (Figure S3). It is well-known that highly ionic ligands form very stable complexes with proteins in the gas-phase.^34^

### CIU of EF-Tu

Following detection of the EF-Tu complexes, we next sought to study the stability of their compact structures using CIU. The [M + 12H]^12+^ ions of apo-EF-Tu, EF-Tu·GDPNP, and EF-Tu·GDPNP·ENX complexes were quadrupole isolated and subjected to increasing collisional activation in the “trap” region of the instrument, and induced ion unfolding was monitored in the TWIMS cell (Figure S4). Collision voltages were varied over the range 30–60 V, corresponding to a laboratory frame collision energy (*E*_lab_) of 360–720 eV. At the lowest *E*_lab_ value, apo-EF-Tu exhibited a corrected PA to TM ^TW^CCS_N2→He_ of 33.8 ± 0.3 nm^2^, EF-Tu·GDPNP a ^TW^CCS_N2→He_ of 34.7 ± 0.6 nm^2^, and EF-Tu·GDPNP·ENX a ^TW^CCS_N2→He_ of 35.6 ± 0.4 nm^2^. Interestingly, the addition of each ligand resulted in an increase in ^TW^CCS_N2→He_, which is at odds with observations in solution, where GDPNP and ENX each induce transition to a more compact conformation.

**Figure 2:**
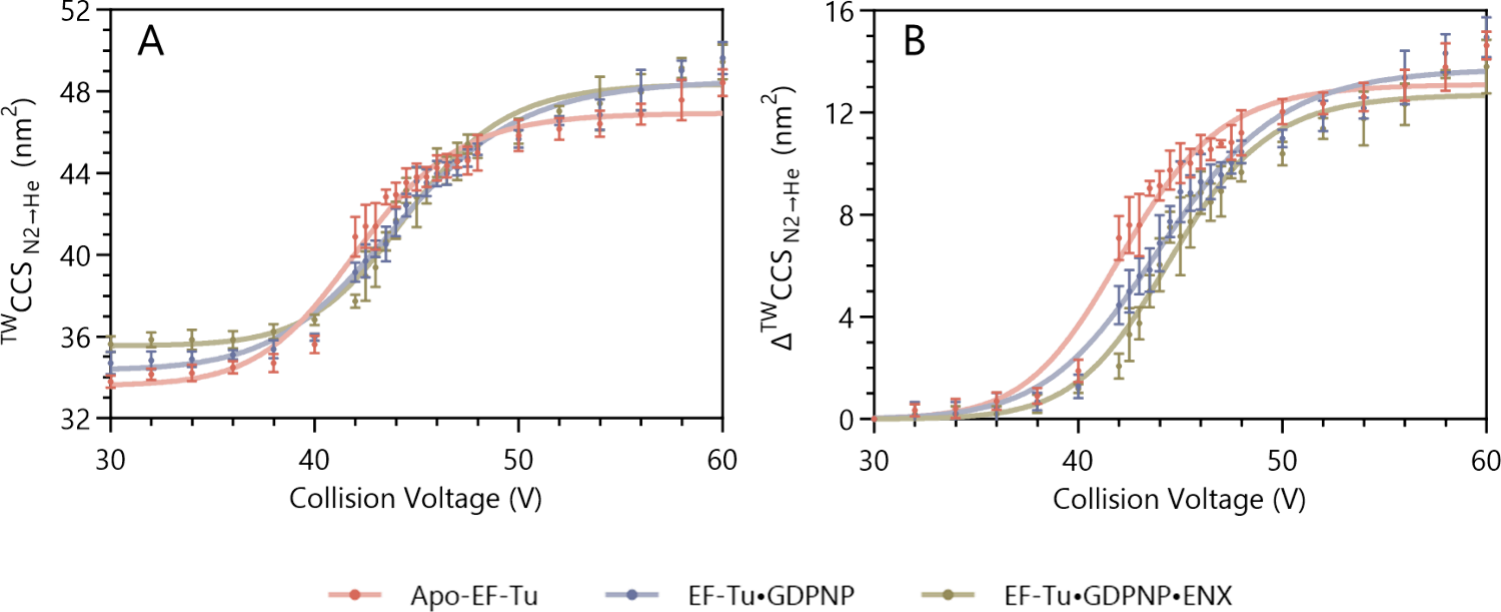
(A) ^TW^CCS_N2→He_ of the three EF-Tu species as a function of the collision voltage applied. Each species undergoes a single unfolding event between 40 and 50 V, with the degree of unfolding being dependent on the presence of ligands. (B) Data shown in (A) converted to ΔCCS. Data are an average of five independent repeats and plotted as mean values alongside their standard deviations, with four-parameter logistic curve fit.

Little unfolding of EF-Tu was observed until a collision voltage of approximately 38–40 V was applied, at which point the protein began to unfold significantly, with the major transition occurring between 40 and 50 V (Figure 2A). Above 50 V, only a small degree of additional unfolding was observed. Comparison between behavior of the different complexes of EF-Tu was most easily drawn from a plot of ΔCCS against collision voltage (Figure 2B), where ΔCCS represents the increase in ^TW^CCS_N2→He_ relative to the most compact structure of each species (namely, at a collision voltage of 30 V, i.e., the initial ^TW^CCS_N2→He_ of the unactivated protein). Apo-EF-Tu clearly exhibited an onset of unfolding at lower collision voltages than either the EF-Tu·GDPNP or EF-Tu·GDPNP·ENX complexes, and in turn, the EF-Tu·GDPNP complex began to unfold at lower voltages than EF-Tu·GDPNP·ENX, showing that the bound ligands increased the stability of EF-Tu’s compact structure.

**Figure 3:**
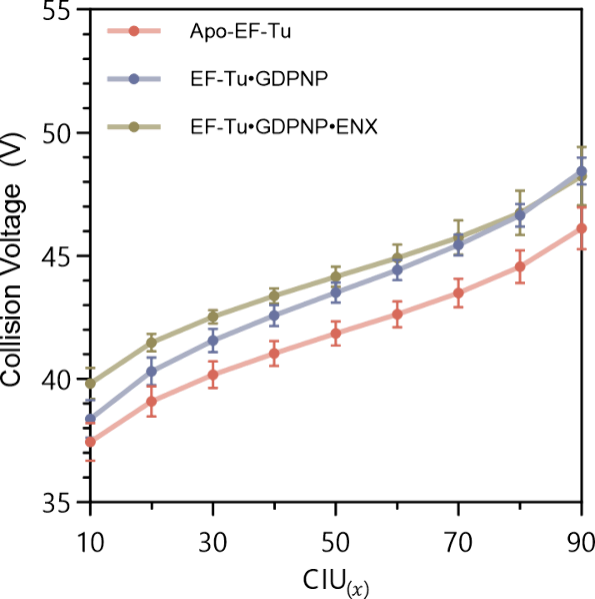
calculated for the three EF-Tu species, with  ranging from 10 to 90. Significant increases in voltages are required to induce equivalent degrees in unfolding of holo-EF-Tu compared to the apo equivalent. Significant increases are also seen in ENX-bound EF-Tu over EF-Tu·GDPNP at  < 40 (*p* ≤ 0.028, ANOVA). Data are an average of five independent repeats and plotted as mean values with their standard deviations.

Quantification of unfolding events during CIU is commonly represented using a CIU_50_ value, which is the voltage, or *E*_lab_ energy, required to induce 50% of the maximal unfolding observed in an unfolding transition. Determination of these values for the three EF-Tu species studied gave CIU_50_ for apo-EF-Tu as 40.3 ± 0.8 V, for EF-Tu·GDPNP as 43.2 ± 0.5 V, and for EF-Tu·GDPNP·ENX as 43.8 ± 1.0 V. Both complexes showed a statistically significant (*p* ≪ 0.05, ANOVA) increase in CIU_50_, and hence stability, over the apo protein, but the difference due to the presence of bound ENX over GDPNP alone was not significant. Given that ENX can dissociate from the EF-Tu·GDPNP·ENX complex, we postulated that this mechanism may be responsible for the modest (insignificant) additional stability afforded by this ligand. Programmed collisional activation revealed that a voltage of 42 V was sufficient to eject 50% of ENX from the [M + 12H]^12+^ ion of the ternary complex (Figure S5), which was very close to the above CIU_50_ values, and meant that a significant amount of dissociation occurred with unfolding. Since the use of CIU_50_ is arbitrary, we examined voltages required to induce , where  is a varying proportion of maximal unfolding (Figure 3). In cases where  ≤ 40%, the EF-Tu·GDPNP·ENX complex did, indeed, show significant stabilization of the compact form over EF-Tu·GDPNP without the bound antibiotic, but this difference was lost at higher collision voltages. This finding was consistent with the hypothesis that dissociation of ENX led to an apparently insignificant effect of this ligand on EF-Tu stabilization when viewed from the perspective of a CIU_50_ value. In cases where ligand dissociation occurs at similar energies to major unfolding, the use of CIU_50_ may mask stabilizing events, and we recommend use of , where  is sufficiently low to show discrimination.

### MD Simulation of EF-Tu

MD simulation was performed on open and closed conformations of the EF-Tu structure taken from PDB coordinates 1EFC and 2BVN, respectively. Ligands were removed to give the apo protein in each conformational form. The holo open form was generated by the reintroduction of GDP, while the holo closed form included GDPNP and ENX. Structures were equilibrated for 50 ps at 298 K by gas-phase MD simulation as described in the Experimental Section. The apo open structure gave a theoretical CCS_CALC_ of 33.1 ± 0.3 nm^2^ while the closed apo CCS_CALC_ was 29.7 ± 0.1 nm^2^. Comparison with the experimental value for apo-EF-Tu of 33.8 ± 0.3 nm^2^ gave a good agreement with the open structure. The theoretical CCS_CALC_ values for the holo proteins were not in such good agreement with experiment, however. Values for the holo open and holo closed were 32.8 ± 0.2 and 30.6 ± 0.0 nm^2^, both of which were considerably smaller than the experimental values (*vide supra*). These results suggest that, under the experimental conditions employed, the IMS measurements did not reflect the change to more compact conformation seen in the crystal structures and maintained in the gas-phase simulations, when GDPNP and ENX bound to the protein. Instead, the presence of any ligand tended to increase the CCS of the complex when compared to the apo protein, even when the change in mass was taken into account in the CCS calibration. This departure from behavior in the condensed phase may be due to the absence of water from the complex or the deposition of a net 12+ charge on the protein ion during the electrospray process.

**Figure 4:**
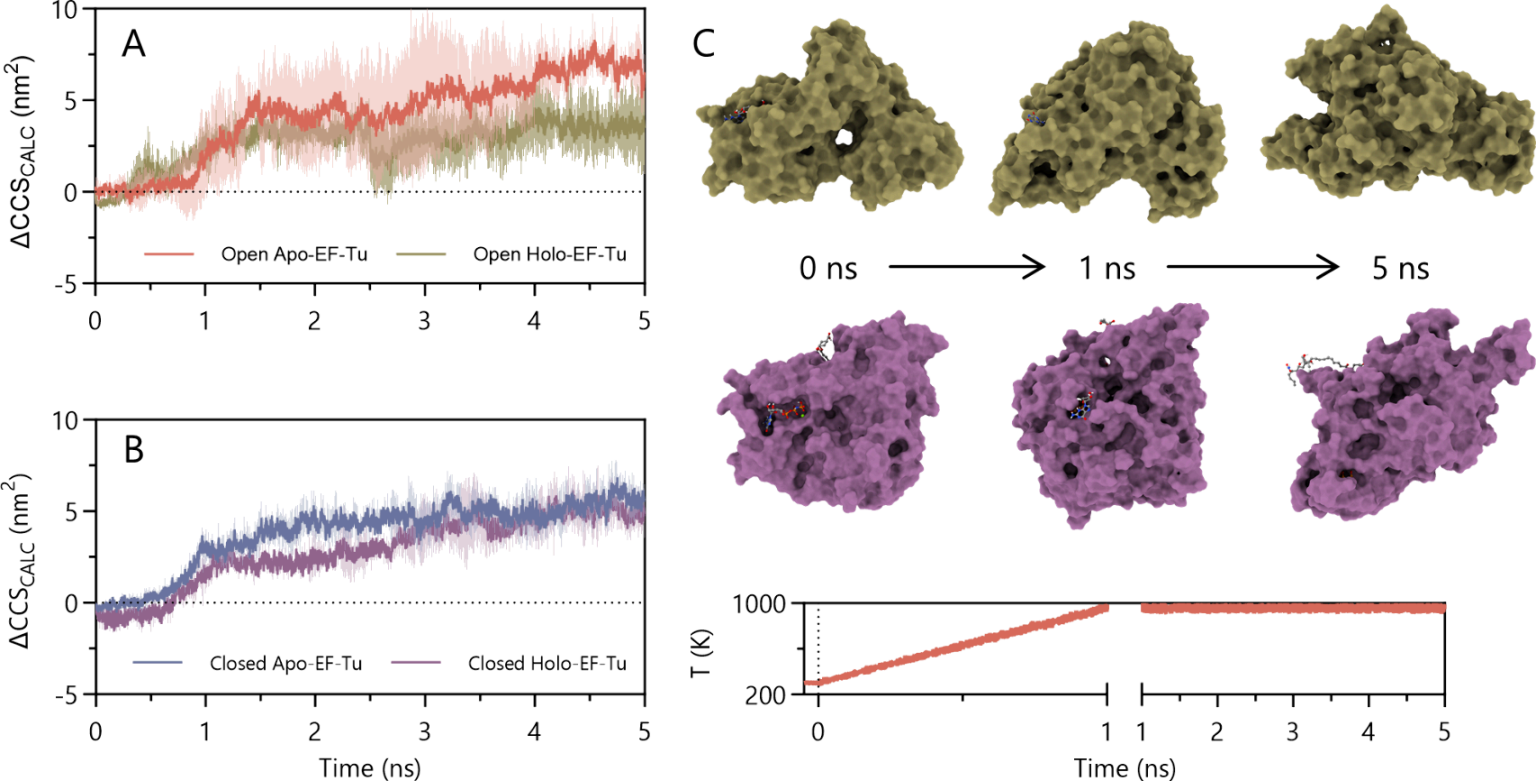
(A) CCS_CALC_ of EF-Tu, initially in the open conformation, over a 5 ns molecular dynamics simulation, with (holo) and without (apo) GDP. Addition of GDP to the complex induced a general reduction in CCS_CALC_ during the simulation and therefore an inferred increase in stability of the compact conformation. (B) CCS_CALC_ of EF-Tu initially in the closed conformation under the same simulation conditions, with (holo) and without (apo) GDPNP and ENX. Addition of the ligands reduced CCS_CALC_ over the first part of the simulation, but their CCS_CALC_ values converged after 3 ns. (C) Representative holo-EF-Tu structures extracted from simulations at the described elapsed times, colored as in (A) and (B). Measured representative protein temperature at the corresponding times is shown. Data is an average of three independent, randomly seeded repeats and plotted as mean values together with their standard deviations.

Following equilibration at 298 K, proteins were next heated to 950 K over 1 ns and maintained at the temperature for 5 ns to induce unfolding (Figure 4). Simulation of the apo and holo open forms showed that the presence of GDP did, indeed, reduce the extent of protein unfolding throughout the MD run (Figure 4A, Figure S7D). Analogous simulations with the closed conformation (Figure 4B, Figures S7B, S7C) revealed that GDPNP and ENX binding delayed unfolding of the holo form but that this effect lasted for the first 3 ns of the simulation only. Toward the end of the simulation, ENX tended to migrate to the outer surface of the protein, but—unlike experiment—did not eject from the protein. GDPNP remained buried within the protein (Figure 4C). In simulations containing GDP, the nucleotide was ejected from the complex in all repeats (Figure S7E). This ejection, occurring between 1.5 and 2.5 ns into the simulation, corresponds to a slight reduction in CCS_CALC_ (Figure 4A). Conversely, no ejection of GDPNP was observed during its respective simulations, while moving locally through the system the nucleotide remained tightly associated with the bound magnesium ion (Figure S7E), whereas this association is lost during GDP ejection. Qualitatively, a reasonable level of agreement between experiment and theory was observed for the gas-phase structure and unfolding of EF-Tu, in particular, stabilization of the compact structure of EF-Tu by GDPNP and ENX binding. There are many reasons why differences in quantitative behavior exist, including the inability of the gas-phase simulations to mimic accurately the collisional activation process and the differences in time frame between MD simulations (ns) and CIU (ms).

## Conclusion

CIU demonstrates that the binding of cofactor and inhibitor ligands to the bacterial elongation factor EF-Tu stabilizes the compact form of the protein in the gas-phase. The effect of ENX is modulated by its dissociation from the complex at energies within the unfolding range. MD simulations performed on the [M + 12H]^12+^ charge state show qualitative agreement with experimental behavior of the ions in terms of added stabilization afforded by ligand binding. There are, however, a number of apparent discrepancies between theory and experiment, in particular, the inability of the gas-phase measurements to show movement of EF-Tu to its known, closed conformation upon binding of GDPNP and ENX. Moreover, while—experimentally—ENX was ejected from the complex at lower energies than GDP and GDPNP, the MD simulation predicted facile dissociation of GDP and no loss of ENX or GDPNP on the simulated time scale.

## Supporting Information Available

Experimental description of EF-Tu expression; select Synapt G1 operating voltages and pressures; residues modified by ChargePlacer; mass spectra; GDP/GDPNP collisional ejection; TWIMS arrival time distributions; calculated ENX EC_50_ value; ChargePlacer benchmarking and protonation sequences; additional molecular dynamics analyses (PDF)
