# Supplementary material for: Native ESI-MS and Collision-Induced Unfolding (CIU) of the Complex between Bacterial Elongation Factor-Tu and the Antibiotic Enacyloxin IIa
Source: J Am Soc Mass Spectrom. 2024 Jun 3;35(7):1490–6. doi: 10.1021/jasms.4c00087 (PMC11228974; doi:10.1021/jasms.4c00087)
Supplement: Supplementary file 1 — js4c00087_si_001.pdf [file js4c00087_si_001.pdf]

Supplementary Information

Native ESI-MS and Collision Induced Unfolding (*CIU*) of the Complex between Bacterial Elongation Factor-Tu and the Antibiotic Enacyloxin IIa

Cameron Baines[1], Jacob Sargeant[2], Christopher D. Fage[2§],Hannah Pugh[2‡] Lona Alkhalaf[2], Gregory L. Challis[2,3,4,5] and Neil J. Oldham[1]*

1. School of Chemistry, University of Nottingham, University Park, Nottingham, NG7 2RD, UK
2. Department of Chemistry, University of Warwick, Coventry, CV4 7AL, UK
3. Warwick Integrative Synthetic Biology Centre, University of Warwick, Coventry, CV4 7AL, UK
4. Department of Biochemistry and Molecular Biology, Biomedicine Discovery Institute, Monash University, Clayton, Victoria 3800, Australia
5. ARC Centre of Excellence for Innovations in Peptide and Protein Science, Monash University, Clayton, Victoria 3800, Australia

*Author for correspondence:

Neil J. Oldham

Email: neil.oldham@nottingham.ac.uk

Tel. +44 (0)115 951 3542

§ Current address: Universite Paris-Saclay, Institute for Integrative Biology of the Cell, Gif-sur-Yvette, Île-de-France, 91190, France

‡ Current address: The Francis Crick Institute, London, NW1 1AT, UK

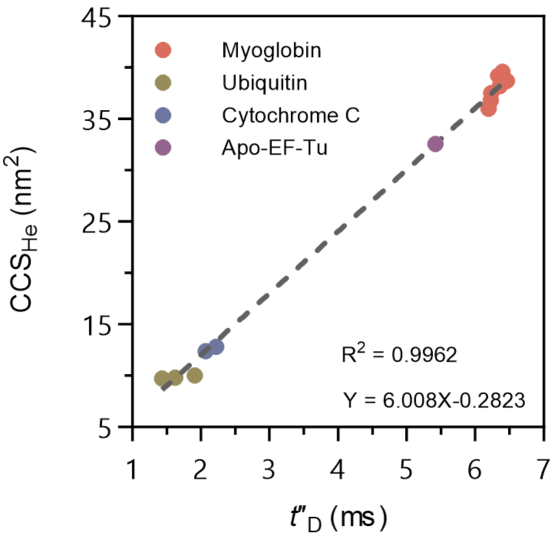

Figure S1: Final IMS calibration plot. Calibrant cross sections (CCS_{He}, converted to nm^2) plotted as a function of a corrected drift time (t''_D, ms) where t''_D represents the experimentally recorded drift time corrected for *m*/*z* dependent delay. From which experimentally obtained EF-Tu drift times can be correlated to $^{TW}CCS_{N2\rightarrow He}$). Calibrants used were denatured myoglobin, native cytochrome C and native ubiquitin, all at a final concentration of 5 μM. Calibrants used bracket native apo-EF-Tu in CCS_{HE}).

Table S1: General instrument conditions used on the Synapt G1 HDMS in both native and IMS experiments. In each experiment the emitter *x*,*y*,*z* stage position was altered in order to give the greatest signal.

Instrument Parameters	Native	Ion Mobility
Capillary	1.3 KV	1.4 - 2.2 KV
Sampling Cone	80 V	80 V
Extraction Cone	5 V	5 V
Collision	10 V	30 - 60 V
Trap Bias	5 V	12 V
Trap T-Wave	300 m/s	300 m/s
IMS T-Wave	300 m/s	280 m/s
Transfer T-Wave	248 m/s	200 m/s
Backing Pressure	4.06E+00	4.07E+00
Source Pressure	2.66E-03	2.68E-03
Trap Pressure	1.84E-02	2.98E-02
Mobility Cell Pressure	5.17E-04	4.31E-01
TOF Pressure	1.60E-06	1.96E-06

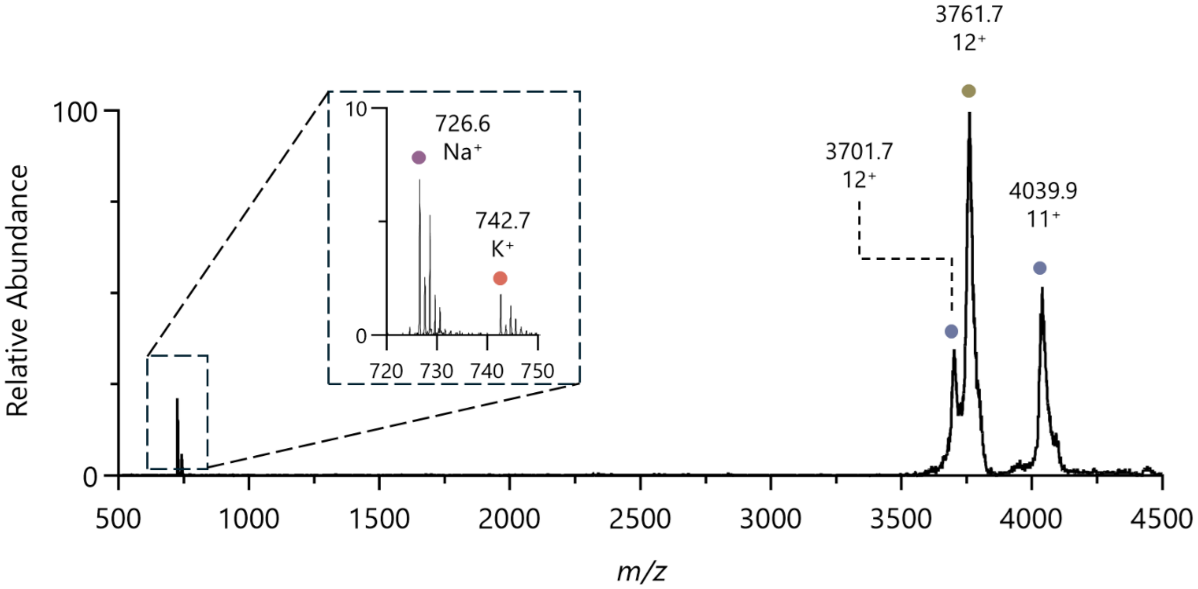

Figure S2: Native mass spectrum of E. coli EF-Tu. Nano-ESI of the protein complex followed by quadrupole isolation of the dominant $[M+12H]^{12+}$ EF-Tu•GDPNP•ENX charge state (green) and collisional activation in the trap cell (collision voltage, 40 V). Following activation, emergence of EF-Tu•GDPNP peaks (blue) at $[M+12H]^{12+}$ and $[M+12H]^{11+}$charge states (3701.7 and 4039.9 *m/z* respectively) can be seen alongside liberated enacyloxin IIa (orange) predominantly featuring as sodiated and potassiated ions (726.6 and 742.7 *m/z* respectively). A reduction in *m/z* of the emergent $[M+12H]^{12+}$ EF-Tu•GDPNP compared to the inactivated species (Figure 1, *m/z* 3701.7 *vs* 3720.3 respectively) can be attributed to loss of adducting ions during the collisional activation.

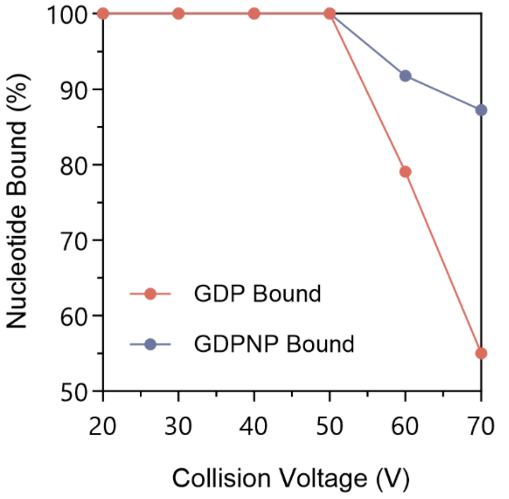

Figure S3: GDP and GDPNP are ejected under standard MS conditions, upon collisional activation of the respective quadrupole isolated nucleotide-bound EF-Tu ions. GDP is more liable for ejection. This ejection is not seen in IM-MS mode.

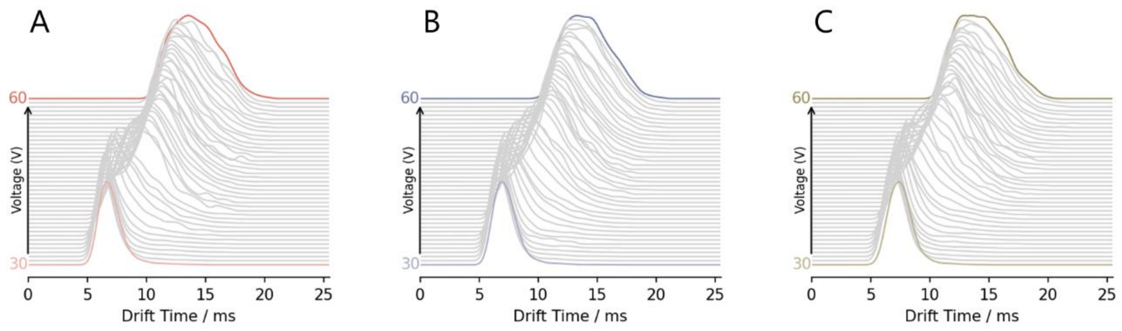

Figure S4: Intensity averaged waterfall plots of the three EF-Tu species in the $[M+12H]^{12+}$ charge state, coloured as in Figure 1 (**A**, apo-EF-Tu; **B**, EF-Tu•GDPNP; **C**, EF-Tu•GDPNP•ENX). Over the collision voltage studied, EF-Tu underwent a single unfolding event. The gradual nature reflects a continuous process of unfolding rather than the adoption of a single defined unfolded state. Data is an average of five independent repeats.

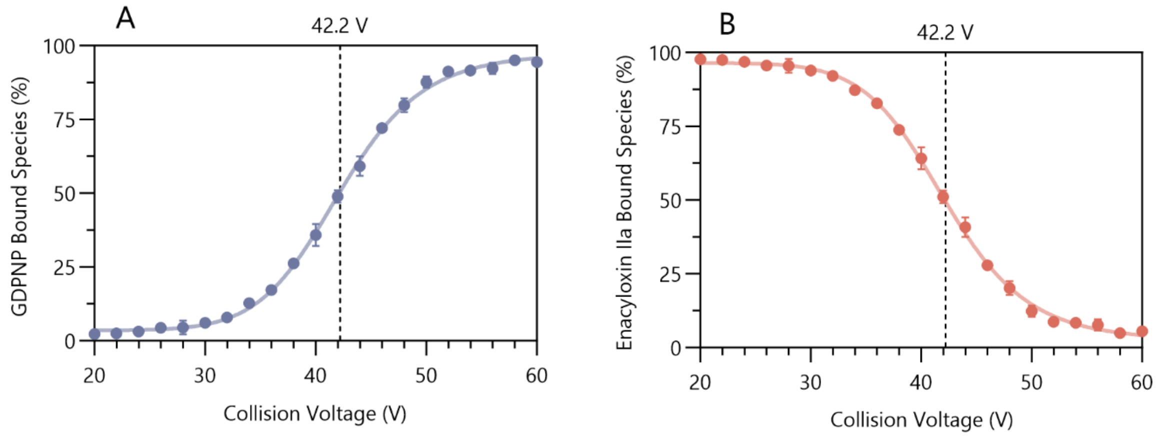

Figure S5: Enacyloxin IIa is ejected from EF-Tu at comparable collision voltages as the calculated CIU_{50} of the protein complex. Emergence of the $[M+12H]^{12+}$, EF-Tu•GDPNP peak was plotted as a proportion of the total protein signal against collision voltage (**A**). The calculated EC_{50} for ENX was found to be 42.2 V. The corresponding inverse (**B**) shows the reduction in $[M+12H]^{12+}$, EF-Tu•GDPNP•ENX as the collision voltage is increased. Data is an average of five independent repeats and plotted as mean values alongside their standard deviation, with four-parameter logistic curves fit.

Table S2: Residues and their respective charge modifications resulting from the ChargePlacer algorithm performed on EF-Tu (PDB: 1EFC). The starting structure has a net charge of -14, with the lowest energy derived proton sequence consisting of a total of 18 residues undergoing deprotonation and 44 being protonated, with a total charge increase of 26, corresponding to an overall net charge of 12+.

Residue	ChargePlacer Modification	Residue	ChargePlacer Modification	Residue	ChargePlacer Modification	Residue	ChargePlacer Modification
Met-1	-1	Asp-110	+1	Asp-167	+1	Lys-264	-1
Glu-4	+1	Glu-118	+1	Glu-180	+1	Asp-267	+1
Lys-10	-1	Arg-124	-1	Asp-182	+1	Glu-273	+1
Asp-22	+1	Lys-137	-1	Glu-184	+1	Glu-285	+1
Lys-25	-1	Asp-139	+1	Lys-188	-1	Lys-295	-1
Asp-48	+1	Asp-142	+1	Glu-191	+1	Lys-300	-1
Asp-51	+1	Asp-143	+1	Asp-197	+1	Glu-316	+1
Glu-55	+1	Glu-144	+1	Glu-202	+1	Arg-334	-1
Glu-56	+1	Glu-145	+1	Asp-208	+1	Glu-346	+1
Lys-57	-1	Glu-148	+1	Lys-209	-1	Glu-349	+1
Arg-59	-1	Glu-151	+1	Glu-216	+1	Asp-355	+1
His-79	-1	Glu-153	+1	Arg-231	-1	Asp-371	+1
Asp-81	+1	Arg-155	-1	Glu-233	+1	Arg-374	-1
Asp-87	+1	Glu-156	+1	Glu-241	+1	Ser-394	+1
Lys-90	-1	Asp-162	+1	Glu-242	+1	**Σ**	**+26**
Asp-100	+1	Asp-166	+1	Glu-250	+1		

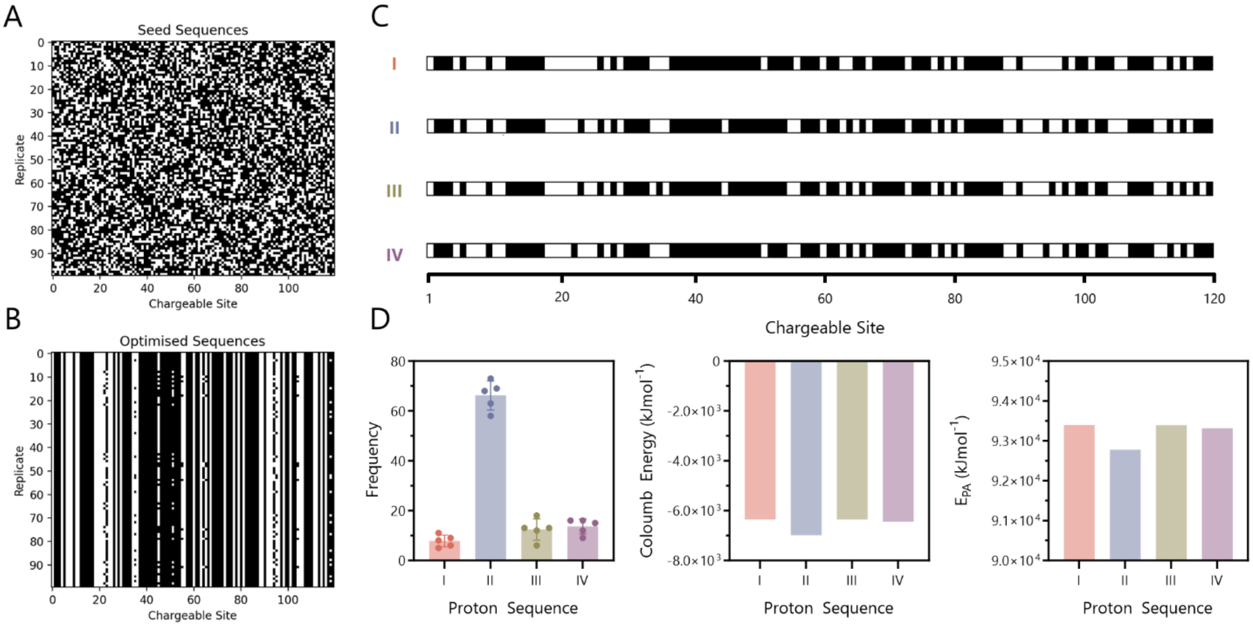

Figure S6: Representative ChargePlacer benchmarking on EF-Tu (PDB: 1EFC), benchmarking consisted of generation of a random input proton sequence seed giving the target net charge of 12+, followed by application of the ChargePlacer algorithm until an energy minimised sequence is reached. This was repeated for 100 input sequences across 5 separate runs. **A**) Representative set of 100 random initial proton sequences across the 120 possible chargeable residues in the EF-Tu sequence. Protonated residues are represented in black. **B**) Each of the input sequences in (**A**) after minimisation using ChargePlacer, the sequences converged to give 4 distinct proton sequences. **C**) The four proton sequence energy minima, sequences were largely consistent with only minor differences between them. Black bars represent proton positions along the120 chargeable sites. **D**) Analyses of the four energy minimised sequences. Sequence II was the most abundant, representing 66.2±5.8 % of the minimised random starting sequences. Sequence II also had the lowest Coulombic energy and proton affinities of the four (-6988.95 and 92778.5 $kJmol^{-1}$ respectively) and was therefore used in the molecular dynamics simulations,

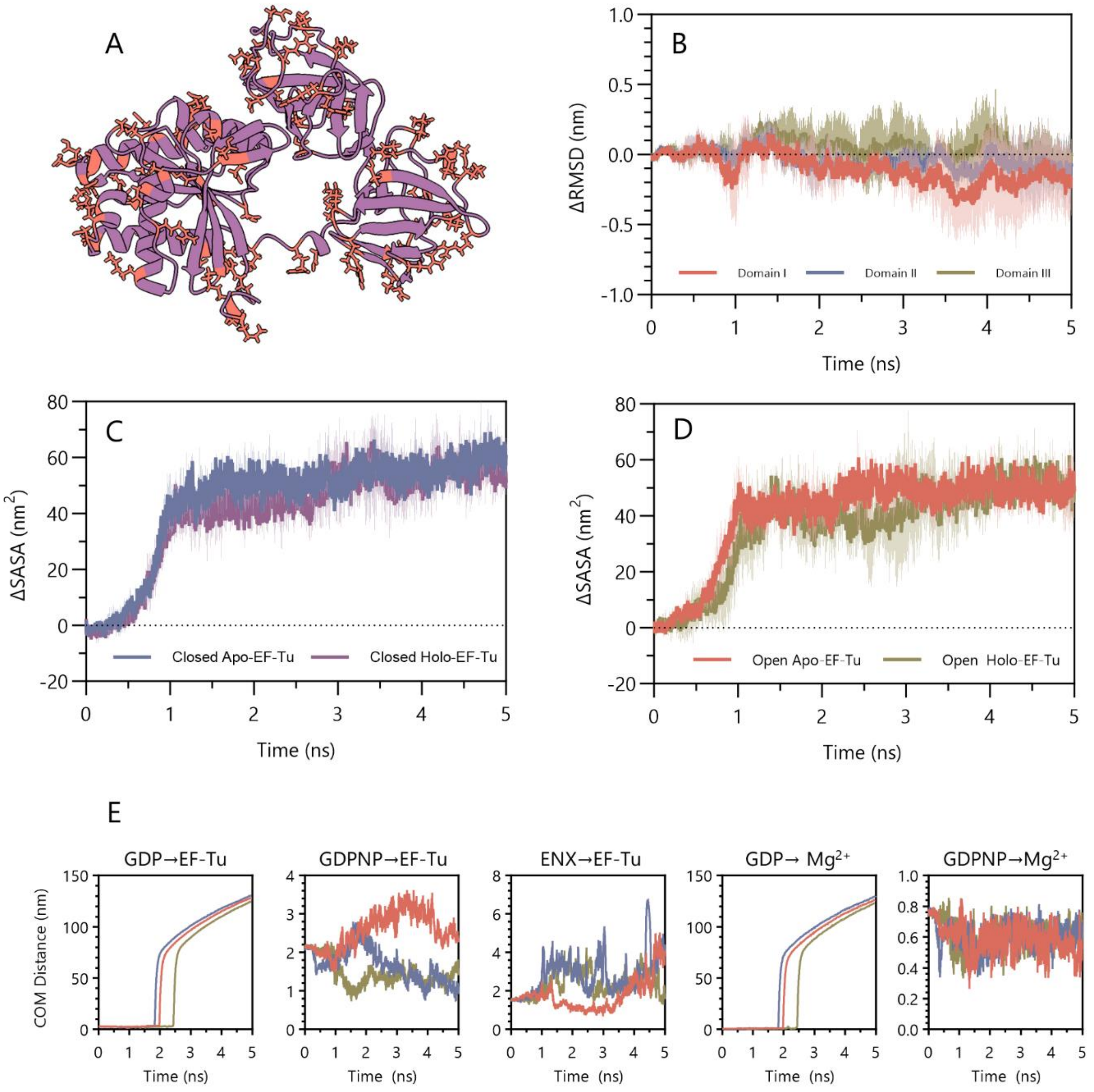

Figure S7: Further molecular dynamics data. (**A**) Spatial location of the 120 chargeable residues identified by ChargePlacer (Table S2) are shown on the apo-open (1EFC, stripped of bound cofactors) EF-Tu structure in orange. (**B**) The addition of GDPNP and ENX to the apo-closed structure of EF-Tu induces a slight increase in stability. Here measured by the change in recorded RMSD compared to the starting structure for each of the three EF-Tu domains upon inclusion of ligands. The greatest increase in stability was found in domain I (nucleotide binding domain), likely due to the presence of GDPNP. (**C**) Measured change in solvent accessible surface area (SASA) over the course of the simulations for closed apo- and holo-EF-Tu. As in Figure 4B, inclusion of GDPNP and ENX initially increase protein stabilisation, as defined here by a comparatively smaller increase in SASA, over the apo-protein up to 2.5 – 3 ns. (**D**) Change in SASA as in (**C**) for open apo- and holo-EF-Tu. Inclusion of GDP reduced the rate of unfolding within the 1 ns ramping period, and initially induced as more compact unfolded state. (**E**) Centre of mass (COM) distances calculated between the stated systems individual corresponding repeats are plotted. GDP was ejected from the open holo-EF-Tu simulations after approximately 2 ns, as shown by the rapid increase in COM distance between GDP, EF-Tu and Mg^{2+}. GDPNP remained tightly associated with the Mg^{2+} cofactor and was no ejected from the system.
